# Association between metabolic syndrome components and chronic kidney disease among 37,533 old Chinese individuals

**DOI:** 10.1007/s11255-021-03013-3

**Published:** 2021-10-21

**Authors:** Lingling Xu, Jin Liu, Dongling Li, Hua Yang, Yang Zhou, Junwei Yang

**Affiliations:** 1 Center for Kidney Disease, Second Affiliated Hospital, Nanjing Medical University, 262N Zhongshan Road, Nanjing, 210003 Jiangsu China; 2 Department of Nephrology, People’s Hospital of Binhai County, Yancheng, 224500 Jiangsu China

**Keywords:** Chronic kidney disease (CKD), Prevalence, Elderly, Metabolic risk factors

## Abstract

**Background:**

Chronic kidney disease (CKD) has become a worldwide health problem among aging populations. However, epidemiological information on Chinese elderly people with CKD is still lacking. This study aimed to investigate the epidemiological features and associated risk factors of CKD in aging population in China.

**Methods:**

In this cross-sectional study, a total of 37,533 individuals aged ≥ 65 years were enrolled in Binhai from January to December 2018. The crude and standardized prevalence of CKD were calculated. Associations of metabolism-related indicators with CKD were examined using univariate and multivariate analyses.

**Results:**

The overall prevalence of CKD was 17.7% (95% confidence interval 17.3–18.1%) in this Chinese elderly population. The prevalence was 17.5% among men (95% CI 17.0–18.1%) and 17.8% among women (95% CI 17.3–18.4%). The mean eGFR was 84.22 (SD ± 12.87) mL/min/1.73 m^2^, with the median value higher for women than for men.

**Conclusion:**

Our study shows a high prevalence of CKD among Chinese elderly population. Aging, pre-HTN, HTN, elevated triglyceride, and FBG were associated with the risk of CKD. More attention should be paid to metabolic diseases to prevent CKD in the elderly.

## Introduction

Chronic kidney disease (CKD) emerges as a growing global public health problem. The prevalence of CKD is estimated to be 10–15% worldwide [1–5]. In 2017, 697.5 million cases of all-stage CKD were recorded, for a global prevalence of 9.1%, and the all-age prevalence of CKD increased by 29.3% from 1990 to 2017 [6]. Outcomes of CKD include not only progression to end-stage renal disease (ESRD) but also multiple associated complications leading to increased morbidity and mortality [7, 8]. The segment of the older population is growing rapidly worldwide. Older people are particularly susceptible to kidney damage from age-related decline in glomerular filtration and chronic disease states, such as diabetes mellitus and hypertension (HTN) [9–11]. The prevalence of CKD is significantly higher in the elderly than that in the general population. Even in developed district, the prevalence of CKD in the elderly is 14.7–21.4% [12–14]. Therefore, it is important to understand the epidemiological characteristics and associated risk factors of CKD in the elderly.

As a high-risk factor of many chronic diseases, metabolic syndrome (MetS) directly affects the progress of the disease. With dramatic economic development and increasingly sedentary lifestyle, the MetS has become common phenomenon in many countries. A cross-sectional study conducted in China shows that the prevalence of MetS was 15.1% [15]. MetS is associated with an increased risk of developing cardiovascular disease (CVD), type 2 diabetes, and related diseases [16]. Recently, many studies found the evidence about the association between MetS and the risk of CKD [15, 17]. MetS was a significant determinant of CKD (OR 1.54; 95% CI 1.28–1.85), and the number of components of MetS has positive impact on the prevalence of CKD [18].

The number of patients with MetS is increasing. However, there are few studies focusing on the epidemiology and risk factors for CKD among the elderly population in China. In the present study, we estimated the prevalence and stages of CKD in a large population aged 65 years and older in eastern China and assessed the risk association between MetS and CKD.

## Materials and methods

### Data sources and participants

Binhai is a district in Jiangsu province, located in the eastern coastal area of China. It was selected because of its universal coverage of free primary care and an integrated electronic health information system. Furthermore, the annual health examination program offered by the local government for Binhai persistent residents is free. The data were extracted using stratified, multistage random sampling from the regional health information system. Finally, a total of 37,533 individuals aged ≥ 65 years in Binhai were enrolled in this study between 1st January 2018 and 31st December 2018.

### Date collection

Urinary protein was measured from a fresh random spot urine sample stored at 4 °C for less than 1 week. Proteinuria was assessed using the urine dipstick test and reported as negative, trace, 1+ , 2+ , or 3+ . We defined proteinuria as trace or greater protein [19]. Blood was collected after an overnight fast of at least 10 h. All blood and urine samples were analyzed at the central laboratory of Binhai Hospital. Serum creatinine was measured using the kinetic rate Jaffe’s method, and estimated glomerular filtration rate (eGFR) was calculated by the CKD epidemiology collaboration creatinine equation (CKD-EPI). The CKD-EPI equation was calculated as GFR (mL/min/1.73 m^2^) = 141 × min (Scr/*κ*, 1)^*α*^ × max (Scr/*κ*, 1)^−1.209^ × 0.993^Age^ × 1.018 (if female) × 1.159 (if Black), where Scr is standardized serum creatinine in mg/dl, *α* is − 0.329 for females and − 0.411 for males, *κ* is 0.7 for females and 0.9 for males, min indicates the minimum of Scr/*κ* or 1, and max indicates the maximum of Scr/*κ* or 1 [20].

### Definition of CKD stage

Renal insufficiency was defined as eGFR less than 60 mL/min per 1.73 m^2^. The CKD stages were categorized based on the classification system established by the Kidney Disease: Improving Global Outcomes (KDIGO) 2012 Clinical Practice Guideline [21]. The CKD stages are defined as follows: Stage 1, proteinuria with eGFR ≥ 90 mL/min/1.73 m^2^; Stage 2, proteinuria with eGFR of 60–89 mL/min/1.73 m^2^; Stage 3, an eGFR of 30–59 mL/min/ 1.73 m^2^; Stage 4, an eGFR of 15–29 mL/min/1.73 m^2^; Stage 5, eGFR < 15 mL/min/1.73 m^2^. Furthermore, patients in Stage 3 can be subdivided as follows: Stage 3a, an eGFR of 45–59 mL/min/ 1.73 m^2^; Stage 3b, an eGFR of 30–44 mL/min/ 1.73 m^2^.

### Definition of metabolic syndrome

We defined central obesity based on a waist circumference greater than 90 cm for men or 80 cm for women [22]. BMI was calculated as weight in kilograms divided by height in meters squared (kg/m^2^), obesity was defined as a BMI of ≥ 28.0 kg/m^2^, and overweight was defined as a BMI between 24.0 and 27.9 kg/m^2^ [23]. HTN was defined as a blood pressure of 140/90 mmHg or more, pre-HTN was defined as systolic blood pressure (SBP) between 120 and 139 mmHg, or diastolic blood pressure (DBP) between 80 and 89 mmHg [24]. According to the American Diabetes mellitus Association 2020 criteria, the baseline fasting blood glucose (FBG) level was categorized into the following three groups: < 100, 100–125, and ≥ 126 mg/dL [25]. According to the International Diabetes Federation (IDF) definition, for a person to be defined as having the metabolic syndrome, they must have: increased waist circumference (Men ≥ 90 cm and Women ≥ 80 cm) plus any two of the following four factors: (1) SBP ≥ 130 or DBP ≥ 85 mmHg or treatment of hypertension; (2) plasma triglycerides (≥ 150 mg/dL) or treated dyslipidemia; (3) fasting HDL cholesterol (men < 40 mg/dL and women < 50 mg/dL); and (4) fasting glucose (≥ 100 mg/dL) or use of anti-diabetic medication [22].

### Statistical analysis

All analyses were done with SPSS Statistics 19.0 (Chicago, IL, USA). Data were presented as the mean ± SD for continuous variables and as proportions for categorical variables. Alanine aminotransferase (ALT) was presented as median with interquartile range (IQR), due to non-normal distribution. Odds ratios (ORs) and prevalence were reported with 95% confidence intervals (CIs). The prevalence was adjusted according to age and sex to represent the total population of elderly by the direct method with the 2010 distribution of the Chinese population [26]. We analyzed the association between CKD and relevant covariates with univariate and multivariate logistic regression models. In the logistic regression models, participants with eGFR < 15 mL/min/1.73 m^2^ were excluded because of different clinical characteristics. Regression estimation was used to deal with the missing data. Two-tailed *t* test *p* value < 0.05 was considered statistically significant.

## Results

### Characteristics of study participants

A total of 37,533 individuals were included in the current analysis. The gender and age distribution of the participants aged ≥ 65 years was 1.07:1 in present study. The average age of the participants in our study was 73.76 ± 5.49 years (minimum 65, maximum 104). The population in this analysis aged 65–74, 75–84, and ≥ 85 years accounted for 63.3%, 31.7%, and 5.0%, respectively.

### The prevalence of indicator of kidney function by CKD stage

**Fig. 1:**
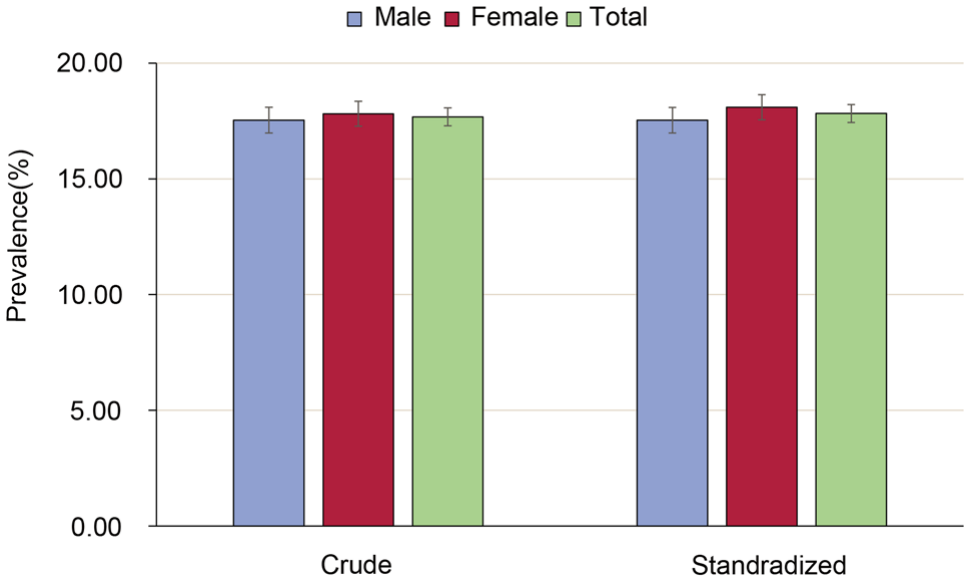
Crude and standardized prevalence of chronic kidney disease. Age- and sex-standardized prevalence was calculated by the direct method with the use of data on the population distribution in China in 2010. I bars indicate 95% confidence intervals

We identified 6,636 (17.7%) CKD cases. The crude prevalence of CKD in older adults in Binhai was 17.7% (95% CI 17.3–18.1%). The prevalence was 17.5% among men (95% CI 17.0–18.1%) and 17.8% among women (95% CI 17.3–18.4%). Furthermore, the age- and sex-standardized overall prevalence of CKD in Chinese older adults was 17.8% (95% CI 17.4–18.2), with a rate of 18.1% (95% CI 17.6–18.6) in women and 17.5% (95% CI 17.0–18.1) in men (shown in Fig. 1). The prevalence of CKD in stages 1, 2, 3 was 7.5%, 4.5%, and 5.4%, respectively, and 0.3% of subjects had severe stage of CKD (stage 4–5). 2,160 (5.8%) participants were classified as chronic renal insufficiency with decreased eGFR. 4997(13.3%) cases had proteinuria, and 521 participants with reduced renal function accompanied by proteinuria (Table 1).

**Table 1:** Prevalence of indicators of kidney function by disease stage

	Kidney function			Proteinuria		CKD
	eGFR (mL/min per 1·73 m^2^)	*n*	Prevalence (%, 95% CI)	*n*	Prevalence (%, 95% CI)	Prevalence (%, 95% CI)
1	≥ 90	14,087	37.5 (37.1–38.0)	2797	19.9 (19.2–20.5)	7.5 (7.2–7.7)
2	60–89	21,286	56.7 (56.2–57.2)	1679	7.9 (7.5–8.2)	4.5 (4.3–4.7)
3	30–59	2039	5.4 (5.2–5.7)	438	21.5 (19.8–23.3)	5.4 (5.2–5.7)
3a	45–59	1644	4.4 (4.2–4.6)	220	13.4 (11.8–15.0)	4.4 (4.2–4.6)
3b	30–44	395	1.1 (1.0–1.2)	218	55.2 (50.5–60.2)	1.1 (0.9–1.2)
4	15–29	86	0.2 (0.2–0.3)	57	66.3 (55.8–76.2)	0.2 (0.2–0.3)
5	< 15	35	0.1 (0.1–0.1)	26	74.3 (58.8–88.2)	0.1 (0.1–0.1)
Total		37,533	100.0	4997	13.3 (13.0–13.7)	17.7 (17.3–18.1)

Proteinuria was defined as trace or greater protein. CKD was defined as eGFR < 60 mL/min per 1·73 m^2^ or proteinuria

*eGFR* estimated glomerular filtration rate, *CKD* chronic kidney disease, *95% CI* 95% confidence interval

### Distribution characteristics of study participants of renal impairment

**Fig. 2:**
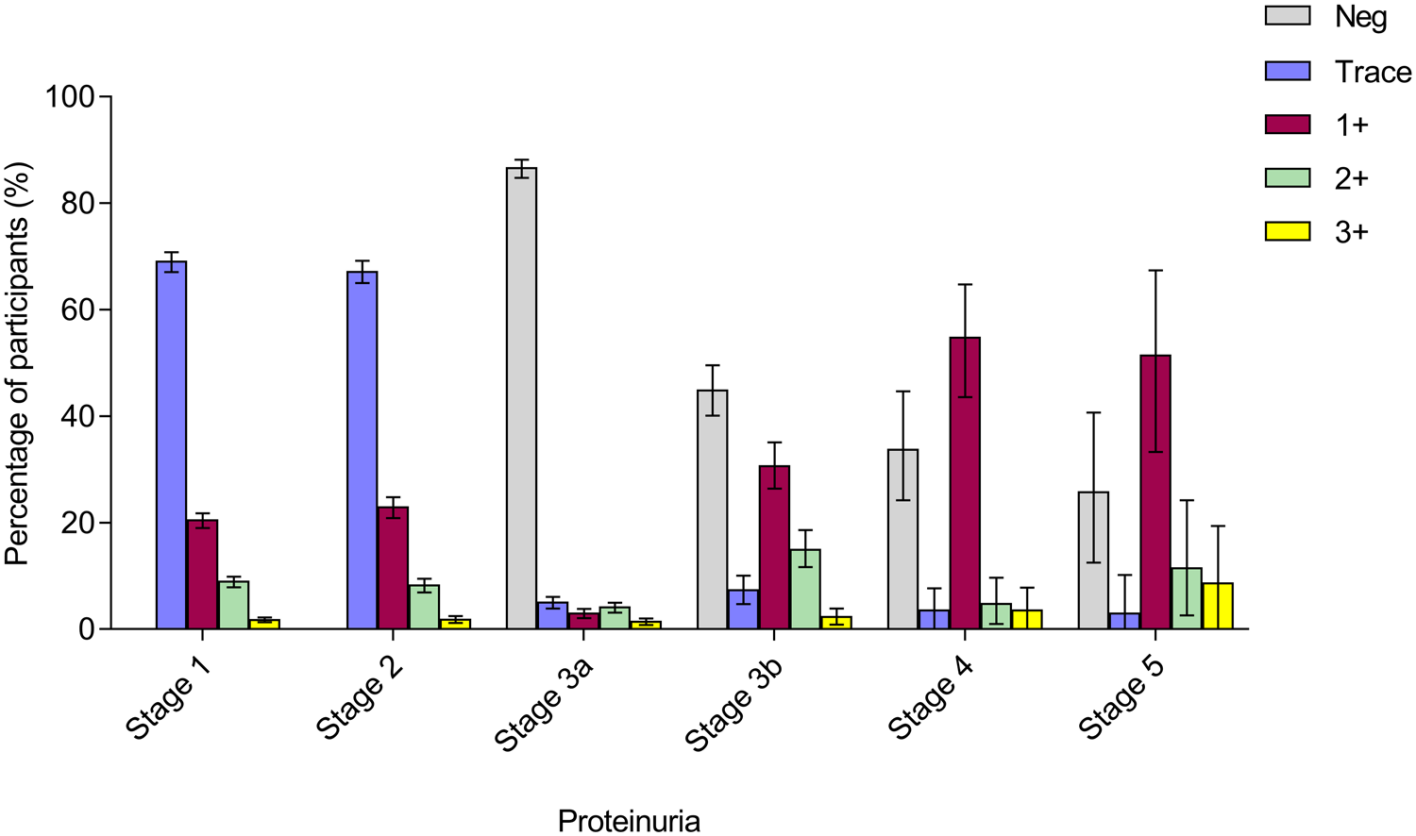
Distribution of proteinuria for participants across different CKD stages. The overall percentages of degree of proteinuria: trace, 1+ , 2+ , and 3+ were 8.4%, 3.2%, 1.4%, and 0.3%, respectively. I bars indicate 95% confidence intervals

**Fig. 3:**
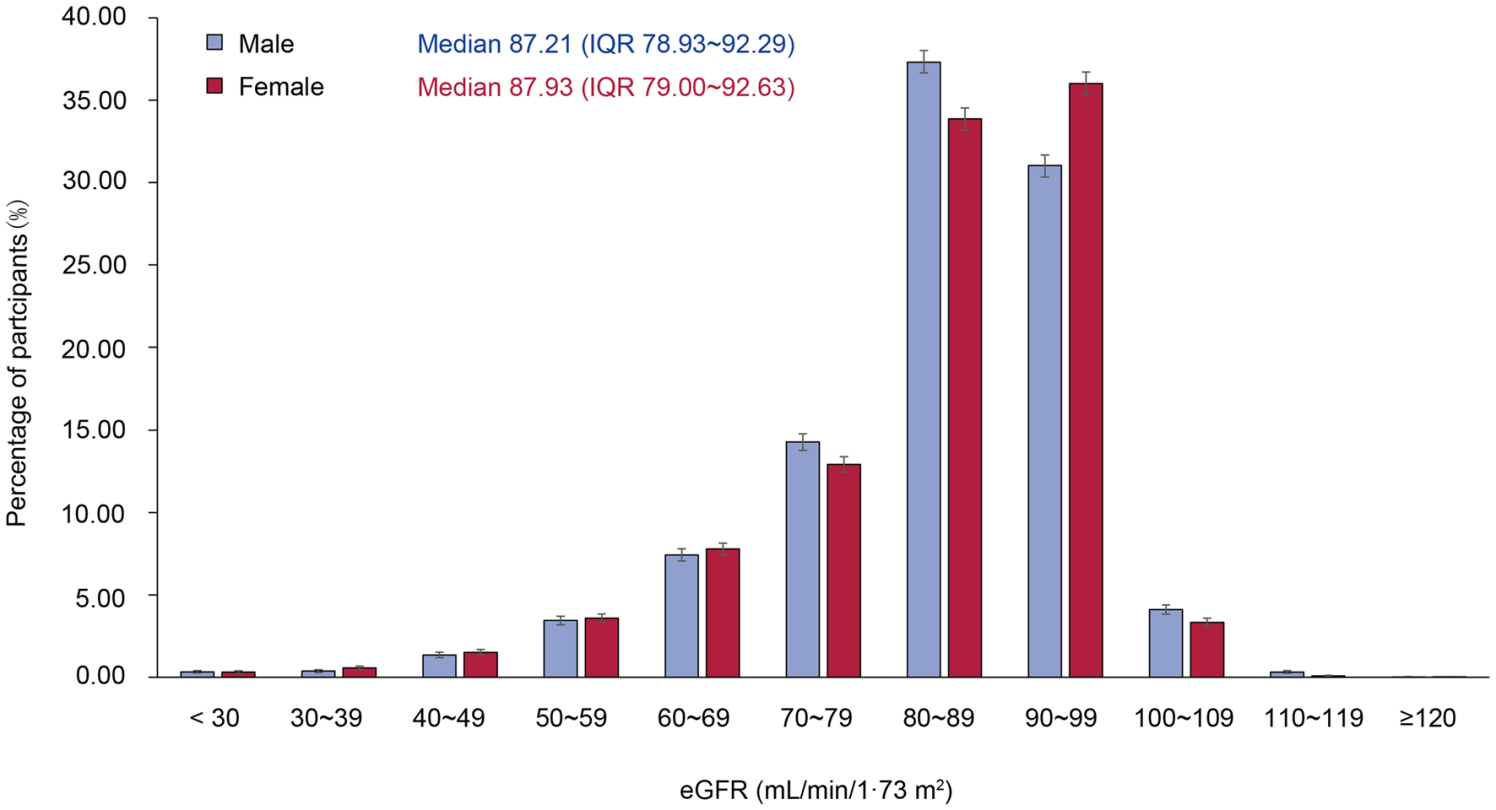
Distribution of kidney function by sex for participants. I bars indicate 95% confidence intervals

13.3% participants had proteinuria. The percentages of degree of proteinuria: trace, 1+ , 2+ , and 3+ were 8.4%, 3.2%, 1.4%, and 0.3%, respectively. In those with proteinuria, most of whom (87.2%) had minimal amount (trace or one plus). Furthermore, 86.6% of participants with CKD stage 3a had no significant proteinuria. The proportion of patients with proteinuria gradually increased with the increase of CKD severity after CKD 3 stage (shown in Fig. 2). There was no statistical difference in the prevalence of proteinuria in women and men (13.2% vs. 13.5%, *p* = 0.403). Mean eGFR in this population was 84.22 mL/min/1.73 m^2^, with the median value higher for women than for men (87.93 vs. 87.21 mL/min/1.73 m^2^, shown in Fig. 3).

The participants who showed indicators of kidney damage were older, especially those with low eGFR. In addition, participants with low eGFR were more likely to be women, who had higher value of BMI, waist circumference, SBP, DBP, Heart rate, total cholesterol, triglycerides, and FBG (Table 2). Among them, 34,457 participants (91.8%) completed the blood routine test. It is remarkable that the hemoglobin concentration of patients with low eGFR is significantly lower than that of participants with proteinuria or non-renal injury group.

**Table 2:** General characteristics of study participants according to indicators of kidney damage

	Participants with no indicators of kidney damage (*n* = 30,897)	Participants with reduced renal function (*n* = 2160)	Participants with albuminuria (*n* = 4997)	Total (*n* = 37,533)
Demographic and clinical data				
Age (years)	73.52 ± 5.32	78.27 ± 6.47	73.62 ± 5.46	73.76 ± 5.49
Male (%)	14,985 (48.50%)	1001 (46.34%)	2447 (48.97%)	18,172 (48.42%)
Physical measurements				
Body mass index (kg/m^2^)	24.67 ± 3.09	24.94 ± 3.20	24.80 ± 3.27	24.69 ± 3.12
Waist circumference (cm)	81.49 ± 7.76	82.26 ± 7.62	81.67 ± 7.89	81.55 ± 7.77
Systolic blood pressure (mmHg)	136.06 ± 18.46	139.54 ± 20.74	137.42 ± 19.13	136.37 ± 18.66
Diastolic blood pressure (mmHg)	79.71 ± 10.24	80.58 ± 11.08	80.14 ± 10.41	79.78 ± 10.30
Heart rate (beats/min)	75.08 ± 8.94	76.19 ± 10.54	75.75 ± 9.58	75.20 ± 9.10
Laboratory data				
Total cholesterol (mg/dL)	200.70 ± 38.94	200.89 ± 43.86	201.51 ± 42.12	200.79 ± 39.50
Triglycerides (mg/dL)	137.50 ± 98.16	159.76 ± 113.87	142.61 ± 113.77	138.97 ± 100.65
ALT (U/L; median [IQR])	17.90 (13.80–24.20)	16.50 (12.80–23.00)	18.00 (13.70–24.70)	17.90 (13.74–24.20)
FBG (mg/dl)	96.79 ± 32.36	102.92 ± 41.93	106.58 ± 46.43	98.28 ± 34.99
Creatinine (mg/dL)	0.75 ± 0.16	1.29 ± 0.59	0.78 ± 0.42	0.77 ± 0.24
eGFR (mL/min/1.73 m^2^)	85.84 ± 9.60	49.55 ± 10.10	84.75 ± 17.26	84.22 ± 12.87
WBC (*10^9/L)	5.97 ± 2.06	6.26 ± 2.05	6.25 ± 2.34	6.01 ± 2.10
Hemoglobin (g/L)	133.19 ± 17.12	128.42 ± 19.91	135.87 ± 19.31	133.27 ± 17.58
Platelet (*10^9/L)	172.02 ± 54.52	172.87 ± 57.71	179.18 ± 60.99	172.96 ± 55.55

Data are *n* (%) or mean (± SD), 521 participants with reduced renal function had proteinuria. ALT was presented as median with interquartile range (IQR), because of non-normally distributed. 34,457 participants completed hemoglobin, WBC and platelet examination

*eGFR* estimated glomerular filtration rate, *WBC*  white blood cell, *FBG* fasting blood glucose, *ALT* alanine aminotransferase

### Associations of CKD with baseline characteristics of study participants

Considering the different clinical characteristics, participants with eGFR < 15 mL/min/1.73 m^2^ were excluded in the logistic regression Analysis. Univariate logistic regression models showed that the risk of CKD was correlated with the increase of age and metabolic-related indicators such as obesity, central obesity, pre-HTN, HTN, increased FBG, elevated triglyceride, and total cholesterol level. Gender was not a risk factor of CKD in the elderly. After adjusted with age, these indicators still showed similar results. Multivariate adjustment confirmed that increase of age, HTN, FBG ≥ 100 mg/dL, and triglyceride levels ≥ 2.26 mmol/L were found to be independent risk factors for CKD. In addition, the *p* value for ORs of the association between pre-HTN, total cholesterol level ≥ 6.21 mmol/L, and CKD were both less than 0.1 in the analysis (Table 3).

**Table 3:** Unadjusted and multivariable-adjusted odds of CKD for general characteristics of study participants

	Participants with chronic kidney disease							
	Prevalence *n* (%)	*P* value	OR (95% CI)	*P* value	OR^a^ (95% CI)	*P* value	OR ^b^ (95% CI)	*P* value
Sex								
Female	3449 (17.8)	NS	Ref		–	–	–	
Male	3187 (17.5)		0.981(0.930–1.035)	NS	–	–	–	
Age								
65–69	1430 (15.1)	< 0.001	Ref		–		Ref	
70–74	2290 (16.0)		1.077 (1.002–1.157)	0.043	–		1.075 (0.999–1.156)	0.052
75–79	1364 (18.0)		1.234 (1.138–1.339)	< 0.001	–		1.239 (1.141–1.345)	< 0.001
80–84	1007 (23.4)		1.718 (1.570–1.881)	< 0.001	–		1.748 (1.595–1.917)	< 0.001
≥ 85	545 (29.3)		2.338 (2.085–2.622)	< 0.001	–		2.401 (2.135–2.701)	< 0.001
BMI								
18.5–23.9	2652 (17.1)	0.013	Ref		Ref		Ref	
< 18.5	114 (18.5)		1.096 (0.891–1.349)	NS	1.030 (0.836–1.270)	NS	1.091 (0.884–1.347)	NS
24.0–27.9	2790 (17.6)		1.035 (0.976–1.097)	NS	1.082 (1.020–1.148)	0.009	1.008 (0.948–1.072)	NS
≥ 28	984 (19.1)		1.144 (1.055–1.240)	< 0.001	1.218 (1.122–1.323)	< 0.001	1.058 (0.968–1.157)	NS
Central obesity								
No	4008 (17.2)	0.002	Ref	0.002	Ref		Ref	
Yes	2616 (18.5)		1.091 (1.033–1.152)		1.134 (1.065–1.208)	< 0.001	1.027 (0.968–1.090)	NS
Hypertension								
No	529 (15.3)	< 0.001	Ref		Ref		Ref	
Pre-HTN	3320 (17.0)		1.134 (1.026–1.253)	0.013	1.123 (1.016–1.241)	0.023	1.093 (0.987–1.210)	0.088
HTN	2776 (19.1)		1.308 (1.181–1.447)	< 0.001	1.297 (1.171–1.436)	< 0.001	1.204 (1.084–1.338)	0.001
FBG								
< 100	4416 (16.1)	< 0.001	Ref		Ref		Ref	
100–125	1164 (19.0)		1.216 (1.132–1.306)	< 0.001	1.224 (1.139–1.315)	< 0.001	1.172 (1.089–1.262)	< 0.001
≥ 126	1052 (26.3)		1.85 (1.712–1.999)	< 0.001	1.875 (1.734–2.207)	< 0.001	1.766 (1.630–1.914)	< 0.001
Triglyceride levels (mmol/L)								
< 2.26	5432 (17.1)	< 0.001	Ref		Ref		Ref	
≥ 2.26	1202 (20.8)		1.267 (1.182–1.359)	< 0.001	1.308 (1.218–1.404)	< 0.001	1.159 (1.076–1.248)	< 0.001
Total cholesterol level (mmol/L)								
< 6.21	5551 (17.4)	0.001	Ref		Ref		Ref	
≥ 6.21	1083 (19.3)		1.136 (1.056–1.221)	0.001	1.147 (1.066–1.235)	< 0.001	1.067 (0.989–1.150)	0.092

Chronic kidney disease was defined as eGFR < 60 mL/min/1.73 m^2^ or proteinuria. Central obesity was defined based on a waist circumference greater than 90 cm for men or 80 cm for women

*FBG* fasting blood glucose; HTN, hypertension; *BMI* body mass index; *Ref* reference group; *NS* not statistically significant, therefore not included in the final model; *OR* odds ratio; *95% CI* 95% confidence interval

^a^Adjusted for age and sex

^b^Multivariate analysis

### Associations of individual component of MetS with CKD

**Fig. 4:**
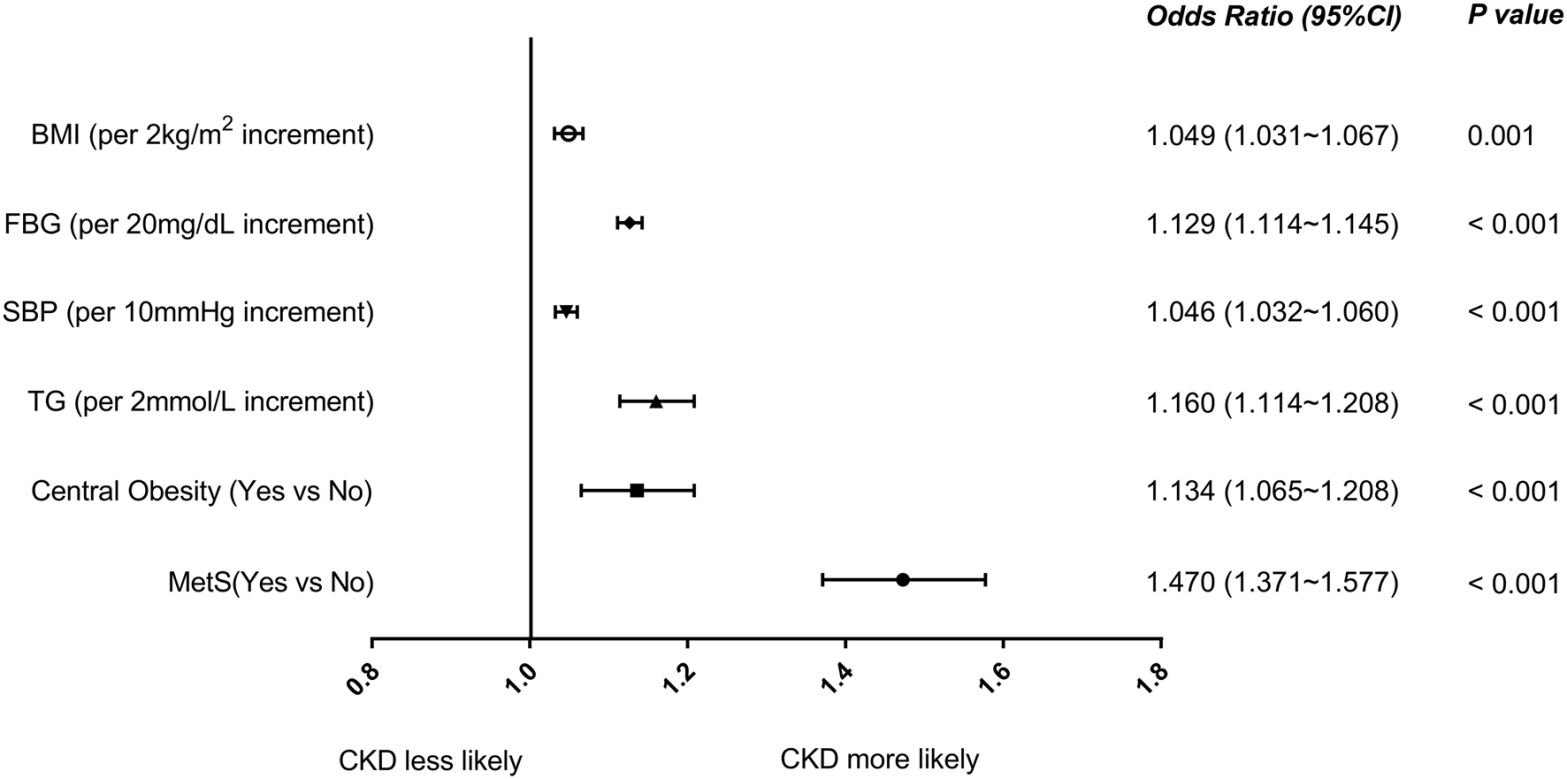
Association between metabolic factors with CKD. Forest plot format shows the odds ratios for metabolic factors (BMI, SBP, FBG, triglyceride, and central obesity), metabolic syndrome, and risk of CKD

**Fig. 5:**
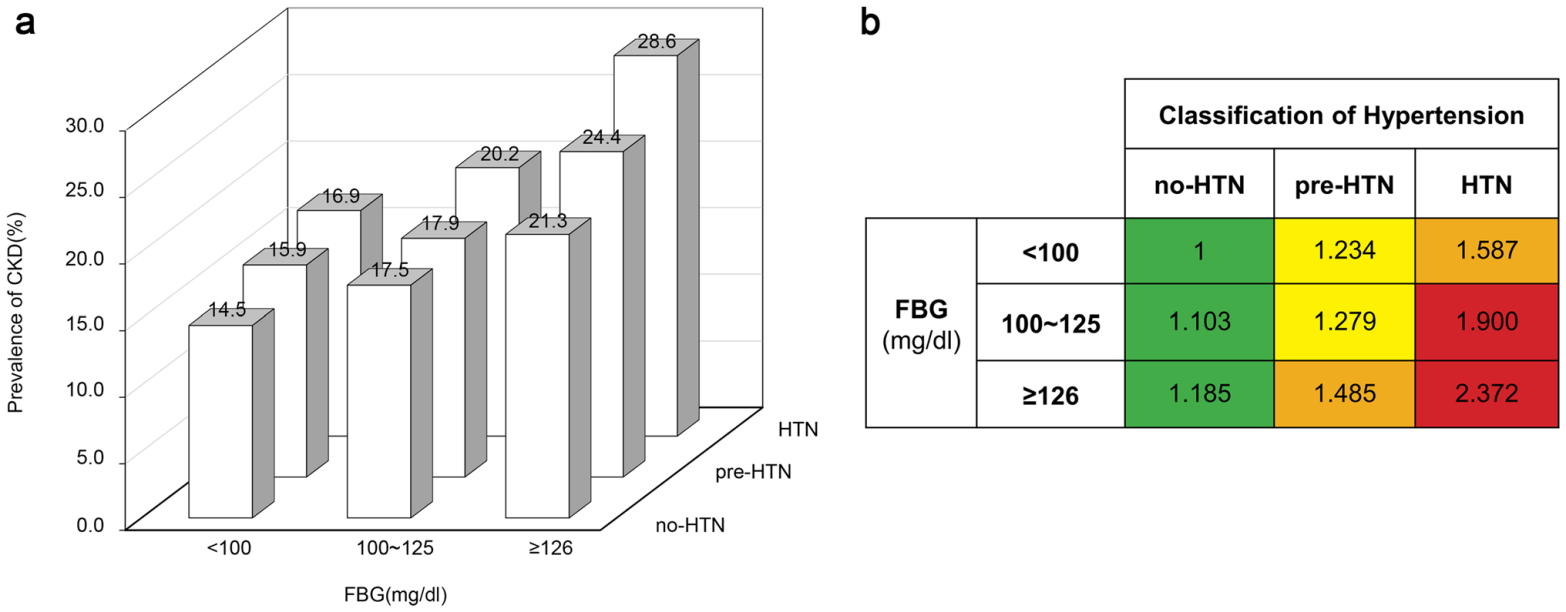
The prevalence and odds ratio for CKD associated with level of FBG and classification of hypertension. The prevalence for CKD are shown in panel **a**. The odds ratio for CKD are shown in panel **b**

To further understand the relationship between these metabolic risk factors and CKD, we stratified these risk factors continuously and evaluated their change trend of age-adjusted ORs for CKD. Our study showed that the risk of CKD was positively correlated with BMI, SBP, FBG, and triglyceride. Of note, patients with MetS had a significantly increased risk of CKD after adjusted with age (shown in Fig. 4). When the population grouped by the FBG (< 100, 100–125, and ≥ 126 mg/dL) and blood pressure (no-HTN, pre-HTN, and HTN). The enhancing effect was found on the prevalence of CKD. There was a positive correlation between prevalence of CKD with FBG and blood pressure, and the CKD prevalence rates in the three groups with gradually rising FBG were 14.5%, 17.5% and 21.3%, respectively (*P* < 0.001). Furthermore, CKD was found in 28.6% of people with FBG ≥ 126 mg/dL and HTN (shown in Fig. 5a).

The risk for CKD was found in the increasing level of FBG and the classification of blood pressure in the elderly (shown in Fig. 5b). In subjects with normal FBG, the ORs for CKD of pre-HTN and HTN were 1.234 and 1.587, respectively, compared with no-HTN. In subjects with FBG ≥ 126 mg/dL, the ORs for CKD of no-HTN, pre-HTN, and HTN compared with those with normal FBG were 1.185, 1.485, and 2.372, respectively. Compared with subjects with normal FBG and blood pressure, those with elevated blood glucose and blood pressure had higher risk of CKD.

## Discussion

In the present study, prevalence of CKD was 17.7%. The effect of gender on CKD was not found. Furthermore, the prevalence of metabolic diseases was significantly high in the population. Older women with higher value of BMI, waist circumference, SBP, DBP, heart rate, total cholesterol, triglycerides, and FBG tend to have lower eGFR. The increase of age and metabolic disorders (HTN, FBG ≥ 100 mg/dL, and triglyceride levels ≥ 2.26 mmol/L) were associated with the risk of CKD. Strong correlations of CKD with elevated FBG and blood pressure were found when the subgroup analysis was performed.

Multiple chronic non-communicable diseases (CNCDs) have become the main disease burden in China, such as diabetes mellitus, HTN, and obesity [27–29]. Aging is one of the major risk factors for CNCDs. In China, the statistics showed that the elderly accounted for 7.0% of the total population in 2000, but it rose to 8.9% in 2010, and there was up to 400 million in 2050 [26, 30]. With the rapid economic development and associated dramatic lifestyle changes, the prevalence of CNCDs in China has increased significantly [31]. In a large-scale population-based survey conducted in 2013 in mainland China, the estimated standardized prevalence of diabetes and pre-diabetes were 10.9% and 35.7%, respectively. It is remarkable that 20.2% and 45.8% of people aged ≥ 60 years were estimated to have diabetes and pre-diabetes [31]. Another nationwide survey conducted between October 2012 and December 2015 indicated that 23.2% (244.5 million) of Chinese people ≥ 18 years of age had HTN, and 41.3% (435.3million) had pre-HTN. Furthermore, the study also showed that the prevalence of HTN was more over 55% among citizens aged ≥ 65 years [32]. Obesity has also become one of most important CNCDs affecting the health of Chinese adults during the past decade. In 2012, prevalence of obesity and overweight (≥ 18 years) was 11.9 and 30.1%, compared with 7.1 and 22.8% in 2002, respectively [33, 34].

The prevalence of CKD was affected by general population with CNCDs, especially the elderly [35]. These results suggested that CNCDs, especially metabolic diseases, should be paid more attention to in the elderly population. A community-based cohort of elderly individuals showed that the annual rate of eGFR decline in men and women without diabetes was 0.8 and 1.4 ml/min/1.73 m^2^, respectively, which increased to 2.1 and 2.7 mL/min/1.73 m^2^ for individuals with diabetes. Michishita et al. [36] found that HTN may be associated with the incidence of CKD in middle-aged and older males. The association between obesity and the risk of CKD has also been confirmed in the previous studies [37]. Our study also draws similar conclusions, the prevalence of CKD was increased with the increasing FBG and with HTN and pre-HTN. Moreover, our study also found that triglycerides, not cholesterol, remained an independent risk factor for CKD. Evidence suggested that decreased glomerular filtration rate and obesity are associated with the risk of CKD [38, 39]. It was reported that elevated triglyceride was significantly associated with a higher risk of arterial stiffness and nephric microvascular damage [40]. In addition to overweight, underweight was also significantly associated with increased risk of CKD. Several studies showed that patients with BMI < 18.5 kg/m^2^ exhibited non-significantly higher events of eGFR decline events in both early and late CKD stages than other BMI groups [41]. It reminds us that CKD is a complex disease that requires individualized nutritional intervention to its treatment.

Unfortunately, even in developed countries, many studies show that the awareness of CKD remains low due to long asymptomatic phase of CKD [42]. CKD is a disease that is amenable to screening. Better management can slow progression of renal dysfunction and multiple associated complications. Fortunately, China launched a health-care reform plan that pledged to provide all citizens with equal access to basic health care with reasonable quality and financial risk protection in 2009 [43]. The local government has conducted annually universal free health examinations for residents in Binhai country since January 2017. It was then gradually integrated with information on population screening, public health surveillance, hospital health information systems, disease management, and other health-care services.

A major strength of the present study is that it was conducted in a large number of samples and explored a new insight regarding the association between certain MetS components and risk of CKD. However, there were several limitations should be stated. First, this was a retrospective cross-sectional analysis; some information may be missing. Second, all covariates were obtained from single measurements; therefore, the reported prevalence of abnormal glucose metabolism, HTN, and pre-HTN might be biased. Finally, the relationship between risk factors and CKD needed further investigation in a cohort study rather than a cross-sectional study. Indeed, we will follow up the current population of this study for several years.

In conclusion, the prevalence CKD among older adults in 2018 was 17.7%, and it was associated with the number of MetS components. The increasing prevalence of multi-metabolic diseases in an aging population would increase the prevalence of CKD. Our results suggest the move to identify these metabolic risk factors earlier and conduct multidisciplinary interventions, particularly lifestyle modifications, which might retard the development of CKD.

## Data Availability

The datasets used during the present study are available from the corresponding author upon reasonable request.

## Abbreviations

CKD: Chronic kidney disease
FBG: Fasting blood glucose
HTN: Hypertension
MetS: Metabolic syndrome
